# YHap: a population model for probabilistic assignment of Y haplogroups from re-sequencing data

**DOI:** 10.1186/1471-2105-14-331

**Published:** 2013-11-19

**Authors:** Fan Zhang, Ruoyan Chen, Dongbing Liu, Xiaotian Yao, Guoqing Li, Yabin Jin, Chang Yu, Yingrui Li, Lachlan JM Coin

**Affiliations:** 1 BGI-shenzhen, Shenzhen, China; 2 Institute for Molecular Bioscience, University of Queensland, Queensland, Australia; 3 Department of Genomics of Complex Disease, School of Public Health, Imperial College, London, UK; 4 Department of Computational Medicine and Bioinformatics, Medical School, University of Michigan, Ann Arbor, USA

## Abstract

**Background:**

Y haplogroup analyses are an important component of genealogical reconstruction, population genetic analyses, medical genetics and forensics. These fields are increasingly moving towards use of low-coverage, high throughput sequencing. While there have been methods recently proposed for assignment of Y haplogroups on the basis of high-coverage sequence data, assignment on the basis of low-coverage data remains challenging.

**Results:**

We developed a new algorithm, YHap, which uses an imputation framework to jointly predict Y chromosome genotypes and assign Y haplogroups using low coverage population sequence data. We use data from the 1000 genomes project to demonstrate that YHap provides accurate Y haplogroup assignment with less than 2x coverage.

**Conclusions:**

Borrowing information across multiple samples within a population using an imputation framework enables accurate Y haplogroup assignment.

## Background

The non-recombining portion of haploid chromosome Y is passed intact from father to son with a mutation rate several times greater than autosomes [1]. As such, patterns of variation in Y are widely used to uncover historical patterns of human migration; are important in genealogical reconstruction and have application in forensic analyses.

The Y Chromosome Consortium (YCC) published a revised Y-chromosome DNA haplogroup tree in 2008, consisting of approximately 600 markers which can be used to characterize 20 major global haplogroups, labeled A-T, as well as sub-classification into a total of 311 haplogroups at the finest level of resolution. Different major haplogroups have been found at high frequencies in different geographical regions, for example the E clade in Africa, and the O clade in Eastern Asia. Particular fine-level haplogroups are found in multiple locations, such as R1a in Eastern Europe, South Asia and Central Asia, indicating migration of R1a from Eurasian Steppes to the new world. The C3 haplogroup, found at high frequency throughout Asia is commonly interpreted as genealogical remnants of the empire of Genghis Khan [2].

Y haplogroup assignment has traditionally been carried out by targeted genotyping using a combination of short tandem repeat typing, multiplex PCR and mini-sequencing [3,4], often using a hierarchical strategy in order to first refine the major haplogroup, and subsequently genotype markers within that haplogroup which illuminate finer levels of resolution. Such a procedure requires substantial amount of wet-lab analysis, requires stringent replication and quality control to eliminate errors which can arise due to the limited amount of information collected at each step. More recently, personal genetic companies have included specific Y chromosome markers on custom genotyping arrays [5,6]. Nevertheless, the resolution available from genotyping arrays is limited by markers included on the chip.

High coverage high throughput sequencing has the potential to capture all single nucleotide and insertion/deletion variation, and as such provide near-perfect assignment of individuals to Y haplogroups. One recently published method (AMY-tree) demonstrated the effectiveness of assigning Y haplogroups with high coverage sequence data [7]. As high coverage sequencing of large population samples remains expensive, low coverage population sequencing, in which each individual is sequenced at less than 2x haploid coverage is an attractive alternative, but this will not capture all individual-level variation. AMY-tree, for example, found insufficient information in low coverage genomes from 1000 genomes project for confident haplogroup assignment [7].

We hypothesize that, given the sharing of haplogroups within an ethnically homogenous population, it should be possible to borrow information across individuals within a population in order to improve haplogroup assignment. In this manuscript, we present the YHap tool, which has been designed for assigning haplogroups to low-coverage population re-sequencing data. YHap borrows information across all samples to assign samples to haplogroups probabilistically, thus providing an accurate representation of the inference which can be made from the data collected. YHap is a complete solution and can also be applied to high-coverage sequence data, as well as data from genotyping arrays.

## Methods

We use the set of haplogroups and mutations defined in [8]. We map the forward and reverse primers described in this manuscript to identify the reported strand of the variation in the GRCh37 reference. After strand correction, we identify whether the mutant allele is the equal to the alternative or reference allele, so that we can subsequently work in reference/alternative allele space on the forward strand, consistent with conventional genotype calling schema. Next, we map each mutation to its position on the pre-defined Y phylogenetic tree T. Finally we create a haplogroup matrix **H** of size N_ref_*L where L is the total number of nodes in T (including leaf and internal nodes) and N_ref_ is the number of pre-defined Y markers. Each entry **H**_***i****l*_ *= {***H**_*ilg*_*}* is a probability distribution vector expressing the probability that a sampled individual from the clade below node *j* carries allele g (in this case either the reference or alternate allele). At leaf nodes, this probability vector is either *{0,1}* or *{1,0},* and at internal nodes, it is the proportion of descendant leaf nodes with reference or alternate alleles, respectively.

To assign a sequenced individual to a specific haplogroup, we obtained genotype likelihoods at each putative variant site (inclusive of all markers in H) from chromosome Y VCF files of the 1000 genome project. This results in a matrix G of size N*M where M is the number of sequenced samples, N is the number of putative variants, and **G**_ij_ = {G_ijg_} is a vector of genotype likelihoods. We then generated an augmented H* matrix by adding in extra sites in **G** but not **H** with probability vector H*_il_ *= {0.5,0.5}.* The pipeline is similar for genotype data, except that the genotype likelihoods are taken to be either 1, if **G**_**i,j**_**=**g or 0 otherwise. To illustrate the entries of this matrix, we have included a heatmap of both the standard Y chromosome consortium positions which are polymorphic in the CEU, as well as the full H* matrix trained on CEU data (Additional file 1: Figure S1).

We can calculate the assignment of each individual using

(1) $$P\;\left(G_{.j}|H_{.l}^{*}\right)=\prod_{i=1..N}\sum_{g=\left\{0,1\right\}}P\;\left(G_{i,j}|g\right)*P\;\left(g|H_{\mathrm{il}}^{*}\right)$$

Where $P\;\left(g|H_{\mathrm{il}}^{*}\right)=H_{\mathrm{ilg}}^{*}$ and $P\;\left(G_{ij}|g\right)=G_{\mathrm{ijg}}$

We can then calculate the posterior probability of each haplogroup amongst a set of haplogroups, where prior haplogroup probability distribution P(H^*^_.l_) is set to the uniform distribution,

(2) $$P\;\left(H_{.l}^{*}|G_{.j}\right)=\frac{P\;\left(G_{.j}|H_{.l}^{*}\right)P\left(H_{.l}^{*}\right)}{\sum_{k=1..L}P\;\left(G_{.j}|H_{.k}^{*}\right)\;P\;\left(H_{.k}^{*}\right)}$$

By restricting the set of haplogroups considered in equation (2), YHap can be customized to either only assign to within the major haplogroups (A through to T), or all possible haplogroups at the finest level of classification.

While this model is sufficient for assigning Y haplogroups individually, it does not capture shared information between sequenced samples adequately, particularly for low coverage sequencing. Given that a population sample will share individuals from the same haplogroup, and while none of these individuals may have enough depth at informative Y haplogroup markers, there is enough information across the pooled reads from all samples from the same haplogroup. However, we do not know a-priori which samples can be pooled as coming from the same haplogroup.

In order to pool information between samples, we treat the allele probability distribution {H*_ilg_*}* at markers present in the sequence data but not present as haplogroup markers, as parameters in our model. We update these parameters using expectation maximization, in which we first calculate the posterior probability assigning each sample j to each haplogroup *l* using equation 2, and then update the {H*_ilg_*}* to reflect the average of genotypes assigned to haplogroup *l* at position *i*, weighted by this posterior probability of assignment. In this way, the model learns which alleles are characteristic of the pre-defined haplogroups, and is thus able to more accurately assign individuals which may not have good coverage at those sites, but which show similarity to other individuals across the Y chromosome. The probabilities P(H^*^_.l_) are also updated at each step to reflect the proportion of haplogroups assigned in the population.

## Results

We applied YHap to low-coverage sequencing data generated in the pilot phase of the 1000 genome consortium which were also part of the Hapmap project, including 19 YRI, 16 JPT, 21 CEU and 9 CHB samples [9]. Major Y haplogroups have been previously assigned to these samples as part of the Hapmap project. The average sequencing depth of these samples is 1.67X as described in 1000 genome Y chromosome analysis report. Compared to haplogroups previously obtained from the Hapmap project [10], YHap showed perfect assignment accuracy (Table 1). We also used YHap on the Hapmap combined phase 1,2,3 Y genotype data and obtained the same assignments previously reported with this data.

**Table 1:** Assignment of individuals included in this study to haplogroups

**Pop**	**ID**	**Hapmap**^**1**^	**1KG**^**2**^	**Chip**^**3**^	**NGS**^**4**^	**Pop**	**ID**	**Hapmap**^**1**^	**1KG**^**2**^	**Chip**^**3**^	**NGS**^**4**^	**Pop**	**ID**	**Hapmap**^**1**^	**1KG**^**2**^	**Chip**^**3**^	**NGS**^**4**^
CHB	NA18558	O	N	N1	N1c1c1	CEU	NA06994	HI	I1	I1	I1b1	YRI	NA18501	E3a	E1b1a8a	E1b1a	E1b1a
CHB	NA18561	O	O2b	O2	O2b	CEU	NA07357	R	R1b1b21	R1b1b2	R1b1b2	YRI	NA18504	E3a7	E1b1a	E1b1a	E1b1a
CHB	NA18562	O	O	O3a	O3a3b1	CEU	NA10851	R	R	R1b1b2	R1b1b2	YRI	NA18507	E3a7	E1b1a	E1b1a	E1b1a
CHB	NA18563	O	O2b	O2	O2b	CEU	NA11829	HI	I1	I1	I1b1	YRI	NA18516	E3a	E1b1a	E1b1a	E1b1a
CHB	NA18572	O	O	O3a	O3a3b1	CEU	NA11831	R	R	R1b1b2	R1b1b2	YRI	NA18522	E3a	E1b1a	E1b1a	E1b1a
CHB	NA18603	O	O	O2	O2a1a	CEU	NA11881	HI	I1	I1	I1b1	YRI	NA18853	E3a	E1b1a8a	E1b1a	E1b1a
CHB	NA18605	O	O	O	O3a3b1	CEU	NA11994	R	R1b1b21	R1b1b2	R1b1b2	YRI	NA18856	E1	E1	E1/E2	E1/E2
CHB	NA18608	O	N	N1	N1c1c	CEU	NA12003	HI	I2b	I2b	I2b	YRI	NA18871	E3a	E1b1a8a	E1b1a	E1b1a
CHB	NA18609	O	O	O3a	O3a3b1	CEU	NA12005	R	R1b1b2	R1b1b2	R1b1b2	YRI	NA19098	E3a	E1b1a	E1b1a	E1b1a
JPT	NA18940	D	D2 × D2b1	D2a	D2a	CEU	NA12043	R	R1	R1b1b2	R1b1b2	YRI	NA19119	E3a	E1b1a8a	E1b1a	E1b1a
JPT	NA18943	O	O2b	O2	O2b	CEU	NA12144	R	R1b1b21	R1b1b2	R1b1b2	YRI	NA19138	E3a	E1b1a8a	E1b1a	E1b1a
JPT	NA18944	D	D2b1	D2a	D2a	CEU	NA12154	R	R1	R1b1b2	R1b1b2	YRI	NA19141	E3a	E1b1a8a	E1b1a	E1b1a
JPT	NA18945	O	O	O3a	O2b	CEU	NA12155	R	R1	R1a1	R1a1c	YRI	NA19144	E3a	E1b1a8a	E1b1a	E1b1a
JPT	NA18948	D	D2b1	D2a	D2a	CEU	NA12716	R	R1	R1b1b2	R1b1b2	YRI	NA19153	E3a	E1b1a8a	E1b1a	E1b1a
JPT	NA18952	D	D	D2a	D2a	CEU	NA12750	HI	I1	I1	I1b1	YRI	NA19160	E3a	E1b1a8a	E1b1a	E1b1a
JPT	NA18953	O	O2b	O2	O2a	CEU	NA1760	R	R	R1b1b2	R1b1b2	YRI	NA19171	E3a7	E1b1a	E1b1a	E1b1a
JPT	NA18959	O	O	O3a	O3a3b1	CEU	NA12762	R	R1	R1b1b2	R1b1b2	YRI	NA19200	E3a7	E1b1a	E1b1a	E1b1a
JPT	NA18960	D	D2 × D2b1	D2a	D2a	CEU	NA12812	R	R1	R1b1b2	R1b1b2	YRI	NA19207	E3a	E1b1a	E1b1a	E1b1a
JPT	NA18961	D	D	D2a	D2a	CEU	NA12814	R	R1	R1b1b2	R1b1b2	YRI	NA19210	E3a7	E1b1a	E1b1a	E1b1a
JPT	NA18965	O	O2b	O2	O2b	CEU	NA12872	R	R1b1b2g	R1b1b2	R1b1b2						
JPT	NA18967	D	D2b1	D2a	D2a	CEU	NA12874	R	R1	R	R1b1b2						
JPT	NA18970	D	D2 × D2b1	D2a	D2a												
JPT	NA18971	C	C1	C1a	C3												
JPT	NA18974	C	C1	C1a	C3												
JPT	NA19005	O	O2b	O2	O2b												

*E1b1a was formerly known as E3a.

^1^Hapmap indicates results from Hapmap consortium.

^2^IKG indicates results form 1000 genomes consortium.

^3^Chip indicates results from YHap applied to Hapmap genotype data.

^4^NGS indicates results from YHap applied to 1000 genomes consortium sequence data.

The resolution reported for YHap is the level at which a single assignment achieved greater than 90% posterior probability.

In order to investigate the ability of YHap to assign finer-level haplogroups we compared YHap results obtained at complete resolution (i.e. considering all haplogroup leaf nodes on the pre-defined Y phylogenetic tree) on both Hapmap genotype data and also 1000 genomes low coverage sequence data (Additional file 1: Table S1). We see that there is complete concordance at the major haplogroup level, and there is increasing uncertainty in assignment as the resolution of assignment increases, particularly using dense genotype data. We also observe that accuracy remains high amongst those assignments which YHap assigns high confidence.

We compared YHap’s performance with AMY-tree on the 1000 genome dataset. Firstly we consider those 1000 genomes samples which were also assigned with AMY-tree based on high coverage Complete Genomics data (Additional file 1: Table S2). YHap achieved greater resolution than AMY-tree relative to this benchmark on 4 of 7 samples (correctly identifying R1b1b2 instead of R1; inferring N instead of NO; D2a instead of D2). In 1 of 7 samples Yhap identified E1b1a7 haplogroup, whereas AMY-hap assigned E1b1a8 using both 1000 genomes and Complete genomics data, although in both cases Yhap assigned the same haplogroup on the basis of Hapmap genotype data.

Next, we use Hapmap genotype data as a validation, using results on 65 samples for which we have Hapmap and 1000 genomes sequence data. Yhap achieved greater resolution than AMY-tree in 30 of 65 samples, whereas AMY-tree never had greater resolution than Yhap. As an example, this included Yhap correctly identifying NA12005 as belonging to R1b1b2 haplogroup vs Root for AMY-tree (Additional file 1: Table S3.) AMY-tree identified a haplogroup inconsistent with Hapmap data in 1 of 65 samples (assigning NA11829 to DE instead of I), and YHap identified an inconsistent haplogroup in 2 of 65 samples (assigning both NA18971 and NA18974 to C2 instead of C3, where AMY-tree only assigned haplogroup C).

Finally, in order to investigate the relationships between sequencing depth and assignment accuracy, we randomly downsampled the original bam files from 1000 genome to 0.6X. For simplicity, we chose JPT to run the test. We see that downsampling increases the uncertainty of assignment (Additional file 1: Table S4), but YHap accuracy remains high amongst those assignments which are made with high posterior probability. This demonstrates that as the underlying amount of information decreases, YHap is still able to extract inference and accurately represent the uncertainty of this inference.

The total complexity for the whole procedure is O(N^2^T), conventionally, when using default settings, it will take almost 10 min to locate 10 ~ 20 individuals and approximately 200 Mb memory.

## Discussion and conclusions

We have demonstrated the utility of using low-coverage population sequence data to accurately resolve Y haplogroups at high resolution. This can be achieved via efficiently borrowing information between individuals in the population which have a common Y haplogroup using a probabilistic assignment model. Moreover, we have demonstrated that it is possible to accurately quantify the uncertainty in the haplogroup assignment, such that even for very low coverage sequence data (0.6x) it is possible to make inference of Y haplogroups, but only achieve high certainty for a top-level haplogroup assignment.

Moreover, YHap can inform discovery of new haplogroup markers. Essentially the conditional haplogroup allele probabilities H*_ij_ (which are intialised with probability {0.5,0.5 } which converge to {0,1} or {1,0} represent new mutations which are exclusive to that haplogroup in the population studied, and represent new mutations on the Y haplogroup tree.

YHap currently only incorporates bi-allelic markers, and does not accommodate STR markers, which is a limitation we plan to address in future versions. We also plan to extend Yhap to allow Mitochondrial haplogroup assignment, however, this requires extending the model to incorporate heteroplasmy as an unknown mixture of multiple haplogroups in the same sample.

While Yhap has been designed using low-coverage whole-genome sequence data, given that it only relies on genotype likelihoods, it will also work for capture sequence data. This opens up the possibility of very cost-effective Y chromosome haplogroup analysis on large populations using a custom designed Y chromosome capture array.

### Availability

YHap is available from http://www1.imperial.ac.uk/medicine/people/l.coin/.

## Competing interests

This study is mainly supported by the National Basic Research Program of China (973 program no. 2011CB809201, 2011CB809202, 2011CB809203), the Chinese 863 program (2012AA02A201), the National Natural Science Foundation of China (30890032, 31161130357). LJMC is supported by an ARC Future Fellowship no. FT110100972.

## Authors’ contributions

We developed this software with a joint effort of two research groups, Lachlan Coin’s lab and Yingrui Li’s team, with equal contribution. LC is the senior author for the project design. YL and CY are the senior authors for the data analysis team. LC and FZ wrote the manuscript. LC, FZ and DL participated in software development. RC, YJ, and XY are responsible for data analysis and GL is in charge of information organizing. The authors are grateful to people in 1000 genome consortium and Hapmap consortium for their help in providing validation information. The manuscript has been seen an approved by all authors.

## Supplementary Material

Additional file 1: Table S1 Concordance of Hapmap array data and 1000 genomes sequence data. **Table S2.** Haplogroup assignments comparison using AMY Complete Genomics data based result as golden standard. **Table S3.** Haplogroup assignments comparison using Yhap Hapmap array data based result as golden standard. **Table S4.** Accuracy and certainty of half-coverage. **Table S5.** Accuracy and certainty of downsampling on high-depth sample from 1000 genome project. **Figure S1.** Heatmap representing probability that haplogroup carries non-reference allele on only Y chromosom consortium SNPs. **Figure S2.** Heatmap representing probability that haplogroup carries non-reference allele at all SNPs modelled. Click here for file
