# Supplementary material for: YHap: a population model for probabilistic assignment of Y haplogroups from re-sequencing data
Source: BMC Bioinformatics. 2013 Nov 19;14:331. doi: 10.1186/1471-2105-14-331 (PMC4225519; doi:10.1186/1471-2105-14-331)
Supplement: Additional file 1: Table S1 — Concordance of Hapmap array data and 1000 genomes sequence data. Table S2. Haplogroup assignments comparison using AMY Complete Genomics data based result as golden standard. Table S3. Haplogroup assignments comparison using Yhap Hapmap array data based result as golden standard. Table S4. Accuracy and certainty of half-coverage. Table S5. Accuracy and certainty of downsampling on high-depth sample from 1000 genome project. Figure S1. Heatmap representing probability that haplogroup carries non-reference allele on only Y chromosom consortium SNPs. Figure S2. Heatmap representing probability that haplogroup carries non-reference allele at all SNPs modelled. [file 1471-2105-14-331-S1.pdf]

Suppl table 1: Concordance of Hapmap array data and 1000 genomes sequence data

			Certainty from 1000 genomes sequence data										
			**0**	**0.1**	**0.2**	**0.3**	**0.4**	**0.5**	**0.6**	**0.7**	**0.8**	**0.9**	***sum***
**Certainty from Hapmap Chip data**	**Level 1**	0.9										100 (100)	*100*
		sum										*100*	*0*
	**Level 2**	0.4						1.5 (0)					*1.5*
		0.5											*0*
		0.6										16.9 (100)	*16.9*
		0.7											*0*
		0.8											*0*
		0.9										81.5 (92)	*81.5*
		sum						*1.5*				*98.4*	*0*
	**Level 3**	0.3										16.9 (0)	*16.9*
		0.4						1.5 (0)					*1.5*
		0.5											*0*
		0.6											*0*
		0.7										3.1 (100)	*3.1*
		0.8										1.5 (100)	*1.5*
		0.9			3.1 (0)							73.8 (96)	*76.9*
		sum			*3.1*			*1.5*				*95.3*	*0*
	**Level 4**	0.1										6.2 (0)	*6.2*
		0.2											*0*
		0.3										10.8 (0)	*10.8*
		0.4					12.3 (0)	1.5 (0)		1.5 (0)			*15.3*
		0.5										6.2 (100)	*6.2*
		0.6										3.1 (50)	*3.1*
		0.7										3.1 (100)	*3.1*
		0.8										1.5 (100)	*1.5*
		0.9			3.1 (0)							50.8 (97)	*53.9*
		sum			*3.1*		*12.3*	*1.5*		*1.5*		*81.7*	*0*
	**Level 5**	0.1										12.3 (0)	*12.3*
		0.2					12.3 (0)					4.6 (0)	*16.9*
		0.3										13.8 (11)	*13.8*
		0.4						1.5 (0)		1.5 (0)			*3*
		0.5											*0*
		0.6											*0*
		0.7											*0*
		0.8										23.1 (100)	*23.1*
		0.9			3.1 (0)							27.7 (94)	*30.8*
		sum			*3.1*		*12.3*	*1.5*		*1.5*		*81.5*	*0*

Numbers indicate the percentage of samples which had an assignment with a given lower bound on certainty from 1000 genomes sequence data (horizontal) and Hapmap genotype data (vertical), and different levels. Level 1 indicates main haplogroup assignment (A-T), while levels 2-5 indicate increasing levels of precision. Numbers in brackets indicate the concordance rate between predictions made by sequence and genotype data within each bin.

Suppl Table 2: Haplogroup assignments comparison using AMY Complete Genomics data based result as golden standard

**ID**	**pop**	**level**	**Yhap array**	**Yhap 1000G**	**AMY 1000**	**AMY Complete Genomics**	**Yhap better**	**AMY better**	**Yhap increases resolution**	**AMY-Tree increases resolution**	**Incorrect AMY assignment**	**Inncorrect Yhap Assignment**
NA07357	CEUY	3	R1b	R1b	R1	R1b	TRUE	FALSE	TRUE			
		4	R1b1	R1b1	R1	R1b1	TRUE	FALSE				
		5	R1b1b=0.9	R1b1b	R1	R1b1b	TRUE	FALSE				
		6	R1b1b2=0.9	R1b1b2	R1	R1b1b2	TRUE	FALSE				
NA10851	CEUY	3	R1b	R1b	R1	R1b	TRUE	FALSE	TRUE			
		4	R1b1	R1b1	R1	R1b1	TRUE	FALSE				
		5	R1b1b=0.9	R1b1b	R1	R1b1b	TRUE	FALSE				
		6	R1b1b2=0.9	R1b1b2	R1	R1b1b2	TRUE	FALSE				
NA18501	YRIY	6	E1b1a7=0.5	E1b1a7	E1b1a8	E1b1a8	FALSE	TRUE				TRUE
		7	E1b1a7a=0.5	E1b1a7a	E1b1a8a	E1b1a8a	FALSE	TRUE				
NA18558	CHBY	1	N	N	NO	N	TRUE	FALSE	TRUE			
		2	N1=1	N1=1	NO	N	TRUE	FALSE				
NA18940	JPTY	3	D2a	D2a	D2	D2a	TRUE	FALSE	TRUE			
Total									4	0	0	1

Performance comparison between Yhap and AMY-tree on 1000 genome project low coverage data when using high depth Complete Genomics data based AMY-tree result as golden standard. Yhap increased 4 individuals' resolution, wrongly assigned 1 individual's assignment at level 6. Note that individuals that are given the completely same results according to both approaches are not shown in this sheet.

Suppl Table 3:Haplogroup assignments comparison using Yhap Hapmap array data based result as golden standard

ID	pop	level	Yhap array	Yhap 1000G	KARAFET	AMY Complete Genomics	Yhap better	AMY better	Yhap increases resolution	AMY-Tree increases resolution	AMY assignment relative to array	Inncorrect Yhap Assignment relative to array
NA07357	CEUY	3	R1b	R1b	R1	R1b	TRUE	FALSE	TRUE			
		4	R1b1	R1b1	R1	R1b1	TRUE	FALSE				
		5	R1b1b(0.9)	R1b1b	R1	R1b1b	TRUE	FALSE				
		6	R1b1b2(0.9)	R1b1b2	R1	R1b1b2	TRUE	FALSE				
NA10851	CEUY	3	R1b	R1b	R1	R1b	TRUE	FALSE	TRUE			
		4	R1b1	R1b1	R1	R1b1	TRUE	FALSE				
		5	R1b1b(0.9)	R1b1b	R1	R1b1b	TRUE	FALSE				
		6	R1b1b2(0.9)	R1b1b2	R1	R1b1b2	TRUE	FALSE				
NA11829	CEUY	1	I	I	DE	NA	TRUE	FALSE			TRUE	
		2	I1(0.67)	I1	DE	NA	TRUE	FALSE				
NA11831	CEUY	3	R1b	R1b	R1	NA	TRUE	FALSE	TRUE			
		4	R1b1	R1b1	R1	NA	TRUE	FALSE				
		5	R1b1b(0.9)	R1b1b	R1	NA	TRUE	FALSE				
		6	R1b1b2(0.9)	R1b1b2	R1	NA	TRUE	FALSE				
NA11994	CEUY	3	R1b	R1b	R1	NA	TRUE	FALSE	TRUE			
		4	R1b1	R1b1	R1	NA	TRUE	FALSE				
		5	R1b1b(0.9)	R1b1b	R1	NA	TRUE	FALSE				
		6	R1b1b2(0.9)	R1b1b2	R1	NA	TRUE	FALSE				
NA12005	CEUY	1	R	R	Root	NA	TRUE	FALSE	TRUE			
		2	R1	R1	Root	NA	TRUE	FALSE				
		3	R1b	R1b	Root	NA	TRUE	FALSE				
		4	R1b1	R1b1	Root	NA	TRUE	FALSE				
		5	R1b1b(0.9)	R1b1b	Root	NA	TRUE	FALSE				
		6	R1b1b2(0.9)	R1b1b2	Root	NA	TRUE	FALSE				
NA12043	CEUY	3	R1b	R1b	R1	NA	TRUE	FALSE	TRUE			
		4	R1b1	R1b1	R1	NA	TRUE	FALSE				
		5	R1b1b(0.9)	R1b1b	R1	NA	TRUE	FALSE				
		6	R1b1b2(0.9)	R1b1b2	R1	NA	TRUE	FALSE				
NA12144	CEUY	3	R1b	R1b	R1	NA	TRUE	FALSE	TRUE			
		4	R1b1	R1b1	R1	NA	TRUE	FALSE				
		5	R1b1b(0.9)	R1b1b	R1	NA	TRUE	FALSE				
		6	R1b1b2(0.9)	R1b1b2	R1	NA	TRUE	FALSE				
NA12154	CEUY	3	R1b	R1b	R1	NA	TRUE	FALSE	TRUE			
		4	R1b1	R1b1	R1	NA	TRUE	FALSE				
		5	R1b1b(0.9)	R1b1b	R1	NA	TRUE	FALSE				
		6	R1b1b2(0.9)	R1b1b2	R1	NA	TRUE	FALSE				
NA12155	CEUY	1	R	R	Root	NA	TRUE	FALSE	TRUE			
		2	R1	R1	Root	NA	TRUE	FALSE				
		3	R1a	R1a	Root	NA	TRUE	FALSE				
		4	R1a1	R1a1	Root	NA	TRUE	FALSE				
NA12716	CEUY	3	R1b	R1b	R1	NA	TRUE	FALSE	TRUE			
		4	R1b1	R1b1	R1	NA	TRUE	FALSE				
		5	R1b1b(0.9)	R1b1b	R1	NA	TRUE	FALSE				

	6	R1b1b2(0.9)	R1b1b2	R1	NA	TRUE	FALSE			
NA12760CEUY	1	R	R	Root	NA	TRUE	FALSE	TRUE		
	2	R1	R1	Root	NA	TRUE	FALSE			
	3	R1b	R1b	Root	NA	TRUE	FALSE			
	4	R1b1	R1b1	Root	NA	TRUE	FALSE			
	5	R1b1b(0.9)	R1b1b	Root	NA	TRUE	FALSE			
	6	R1b1b2(0.9)	R1b1b2	Root	NA	TRUE	FALSE			
NA12762CEUY	1	R	R	Root	NA	TRUE	FALSE	TRUE		
	2	R1	R1	Root	NA	TRUE	FALSE			
	3	R1b	R1b	Root	NA	TRUE	FALSE			
	4	R1b1	R1b1	Root	NA	TRUE	FALSE			
	5	R1b1b(0.9)	R1b1b	Root	NA	TRUE	FALSE			
	6	R1b1b2(0.9)	R1b1b2	Root	NA	TRUE	FALSE			
NA12812CEUY	3	R1b	R1b	R1	NA	TRUE	FALSE	TRUE		
	4	R1b1	R1b1	R1	NA	TRUE	FALSE			
	5	R1b1b(0.9)	R1b1b	R1	NA	TRUE	FALSE			
	6	R1b1b2(0.9)	R1b1b2	R1	NA	TRUE	FALSE			
NA12814CEUY	3	R1b	R1b	R1	NA	TRUE	FALSE	TRUE		
	4	R1b1	R1b1	R1	NA	TRUE	FALSE			
	5	R1b1b(0.9)	R1b1b	R1	NA	TRUE	FALSE			
	6	R1b1b2(0.9)	R1b1b2	R1	NA	TRUE	FALSE			
NA18558CHBY	1	N	N	NO	N	TRUE	FALSE	TRUE		
	2	N1	N1	NO	N	TRUE	FALSE			
	3	N1c(0.75)	N1c	NO	N	TRUE	FALSE			
	4	N1c1(0.75)	N1c1	NO	N	TRUE	FALSE			
NA18562CHBY	4	O3a3(0.501)	O3a3	O3a	NA	TRUE	FALSE	TRUE		
NA18572CHBY	4	O3a3(0.501)	O3a3	O3a	NA	TRUE	FALSE	TRUE		
NA18603CHBY	1	O	O	NO	NA	TRUE	FALSE	TRUE		
	2	O2(0.67)	O2	NO	NA	TRUE	FALSE			
NA18605CHBY	4	O3a3(0.501)	O3a3	O3a	NA	TRUE	FALSE	TRUE		
NA18608CHBY	1	N	N	NO	NA	TRUE	FALSE	TRUE		
	2	N1	N1	NO	NA	TRUE	FALSE			
	3	N1c(0.75)	N1c	NO	NA	TRUE	FALSE			
	4	N1c1(0.75)	N1c1	NO	NA	TRUE	FALSE			
NA18609CHBY	4	O3a3(0.501)	O3a3	O3a	NA	TRUE	FALSE	TRUE		
NA18940 JPTY	3	D2a	D2a	D2	D2a	TRUE	FALSE	TRUE		
NA18945 JPTY	2	O3	O2	O3	NA	#####	TRUE		TRUE	TRUE
	3	O3a	O2b	O3a	NA	#####	TRUE			
NA18952 JPTY	1	D	D	DE	NA	TRUE	FALSE	TRUE		
	2	D2	D2	DE	NA	TRUE	FALSE			
	3	D2a	D2a	DE	NA	TRUE	FALSE			
NA18953 JPTY	1	O	O	NO	NA	TRUE	FALSE	TRUE		
	2	O2(0.67)	O2	NO	NA	TRUE	FALSE			
NA18959 JPTY	4	O3a3(0.63)	O3a3	O3a	NA	TRUE	FALSE	TRUE		
NA18960 JPTY	1	D	D	DE	NA	TRUE	FALSE	TRUE		
	2	D2	D2	DE	NA	TRUE	FALSE			
	3	D2a	D2a	DE	NA	TRUE	FALSE			
NA18961 JPTY	1	D	D	DE	NA	TRUE	FALSE	TRUE		

		2	D2	D2	DE	NA	TRUE	FALSE				
		3	D2a	D2a	DE	NA	TRUE	FALSE				
NA18970	JPTY	1	D	D	DE	NA	TRUE	FALSE	TRUE			
		2	D2	D2	DE	NA	TRUE	FALSE				
		3	D2a	D2a	DE	NA	TRUE	FALSE				
NA18971	JPTY	2	C1	C3	C	NA	#####	FALSE				TRUE
NA18974	JPTY	2	C1	C3	C	NA	#####	FALSE				TRUE
NA19171	YRIY	7	E1b1a7a(0.67)	E1b1a7a	E1b1a7	NA	TRUE	FALSE	TRUE			
NA19210	YRIY	2	E1	E1	E	NA	TRUE	FALSE	TRUE			
		3	E1b	E1b	E	NA	TRUE	FALSE				
		4	E1b1	E1b1	E	NA	TRUE	FALSE				
		5	E1b1a	E1b1a	E	NA	TRUE	FALSE				
		6	E1b1a7(0.67)	E1b1a7	E	NA	TRUE	FALSE				
		7	E1b1a7a(0.67)	E1b1a7a	E	NA	TRUE	FALSE				
Total									30	1	1	3

Performance comparison between Yhap and AMY-tree on 1000 genome project low coverage sequence data, using Hapmap project's array data called with Yhap as gold standard. Yhap increased 26 individuals' resolution, wrongly assigned 1 individual's assignment at level 2. AMY-tree wrongly assigned 5 individuals and has no better resolution result. Table displays all samples for which Yhap and AMY-tree produced discordant Y haplogroups which could be adjudicated by the gold standard, which requires the gold standard prediction was made with more than 50% certainty, and agreed with one of the discordant Yhap and AMY-tree assignments. Number in brackets indicates certainty of assignment, which is 1.0 if not specified.

Suppl table 4: Accuracy and certainty of half-coverage

Certainty	%age	Accuracy (%)
0	6.2	0
0.1	12.5	50
0.2	6.2	100
0.3	6.2	100
0.4	6.2	0
0.5	6.2	100
0.6	0	0
0.7	12.5	100
0.8	12.5	100
0.9	31.2	100
Average		81.05

Percentage of calls made with certainty lower bound on half-coverage at top level of assignment, and concordance with Hapmap assignment.

Suppl table 5: Accuracy and certainty of downsampling on high-depth sample from 1000 genome project

Depth	Assignment/Certainty
18X	I1a=0.254;I1b1=0.238;I1c=0.254;I1d=0.254
9X	I1a=0.254;I1b1=0.238;I1c=0.254;I1d=0.254
5X	I1a=0.254;I1b1=0.238;I1c=0.254;I1d=0.254
2X	I1a=0.253;I1b1=0.240;I1c=0.253;I1d=0.253

Here the sample we used is NA12891, one of the trio sample from 1000 genome project of 18X on chrY. The assignment from 1000 genome chrY report is I.

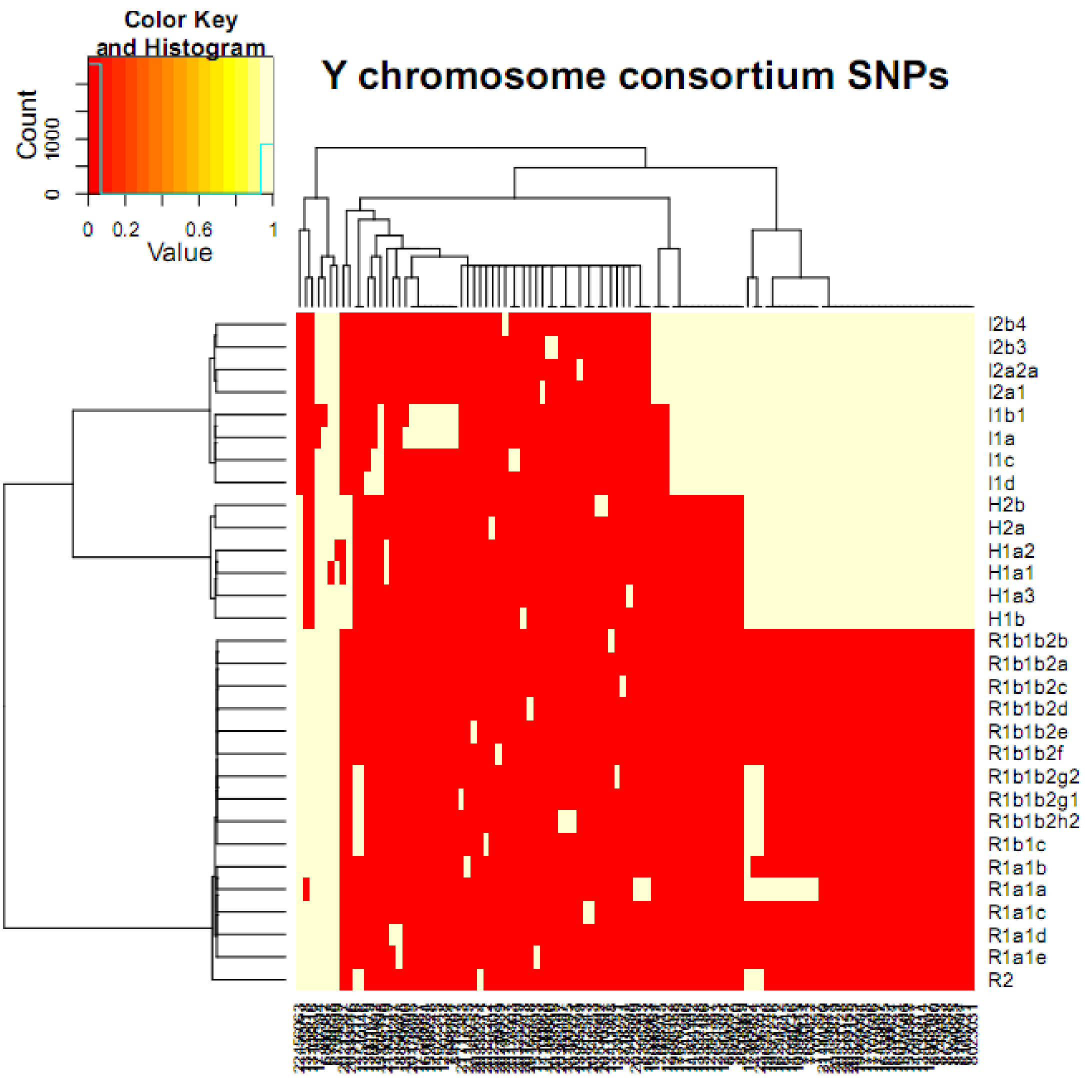

Suppl. Fig 1:

This figure illustrated the heat map of the Y chromosome consortium SNPs, pre-defined markers, used in assignment process. The horizontal axis represents different markers and the vertical axis represents different nodes from YCC tree. Here we only presented CEU population related markers and nodes. Each entry in the map corresponds to the entry in H matrix.

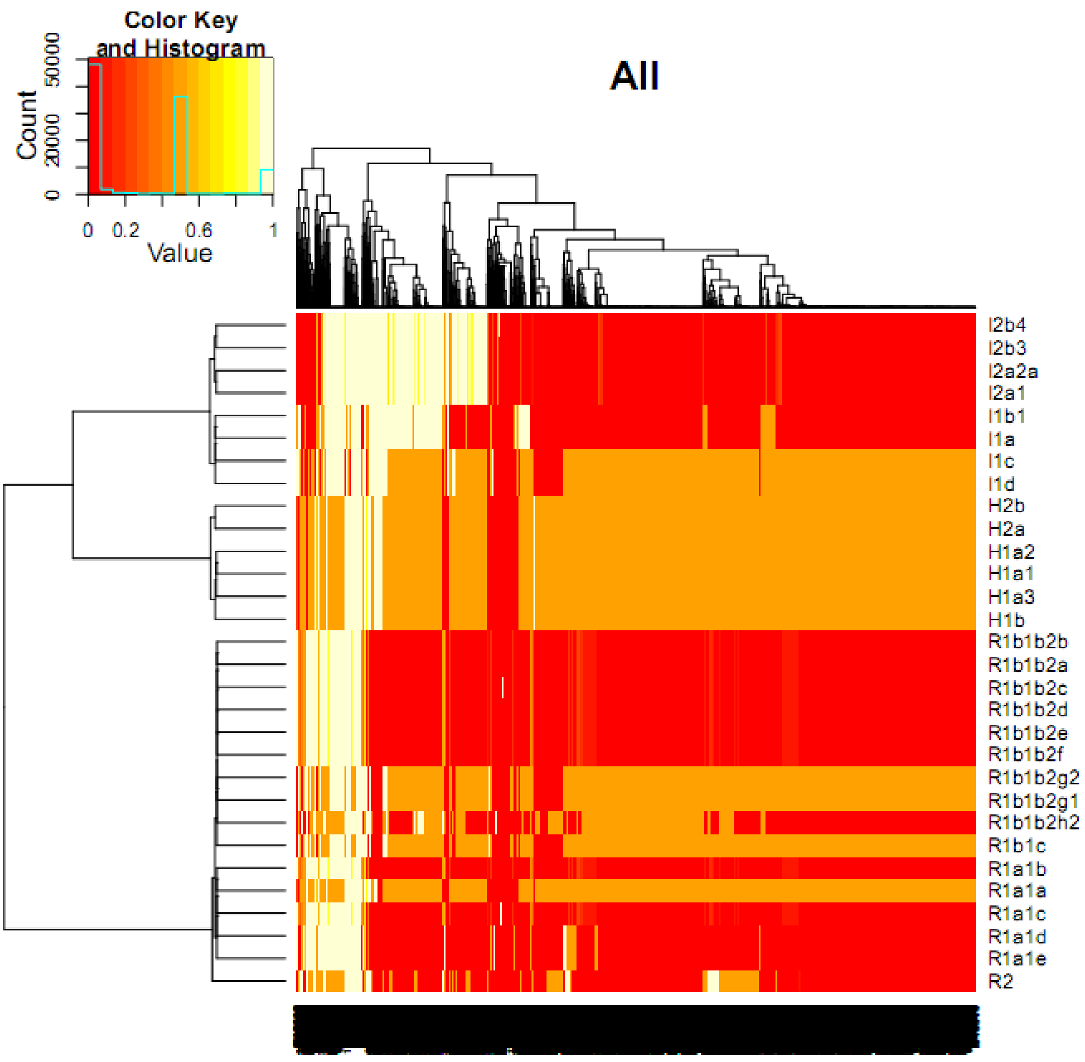

Suppl. Fig 2:

This figure illustrated the heat map of all the markers included in assignment process. The horizontal axis represents different markers and the vertical axis represents different nodes from YCC tree. Here we only presented CEU population related markers and nodes. Each entry in the map corresponds to the entry of augmented matrix, i.e. the H* matrix.
